# Alpha-amylase reactivity and recovery patterns in anhedonic young adults performing a tandem skydive

**DOI:** 10.1371/journal.pone.0204556

**Published:** 2018-09-24

**Authors:** Charlotte Vrijen, Eeske van Roekel, Albertine J. Oldehinkel

**Affiliations:** 1 Interdisciplinary Center Psychopathology and Emotion regulation, University Medical Center Groningen, University of Groningen, Groningen, The Netherlands; 2 Department of Developmental Psychology, Tilburg University, Tilburg, The Netherlands; Public Library of Science, UNITED KINGDOM

## Abstract

**Background:**

Anhedonia (loss of pleasure) is characterized by low responsiveness to rewards and, by virtue of being one of the two core symptoms of depression, by altered responses to stress. We investigated the effect of an acute stress experience (i.e., a tandem skydive) that was expected to elicit both intense fear and intense euphoria in a sample of anhedonic young adults.

**Objective:**

(1) To examine individual differences in alpha-amylase reactivity to and recovery from a tandem skydive in anhedonic young adults; (2) to investigate whether trait depressive and anxiety problems, trait positive affect (PA), i.e., level of pleasure and reward responsiveness, and state anxiety, PA and self-esteem prior to the skydive were associated with alpha-amylase reactivity and recovery patterns; (3) to investigate whether alpha-amylase reactivity and recovery patterns were associated with pre- to post-jump changes in state anxiety, PA, and self-esteem.

**Method:**

Participants were 61 individuals with persistent anhedonia (*M*age = 21.38, 78.7% female), who filled out a baseline questionnaire at the start of the study, and momentary questionnaires (3 times per day) before and after the tandem skydive. Alpha-amylase was measured at four time points by means of salivettes (2 before and 2 after the skydive).

**Results:**

Alpha-amylase reactivity and recovery patterns were highly similar across individuals, although mean levels varied greatly. No associations were found between any of the trait and state measures and reactivity and recovery. Only state self-esteem was affected by the reactivity and recovery patterns, in that individuals who showed high reactivity and low recovery experienced decreases in self-esteem after the skydive.

**Conclusions:**

Alpha-amylase patterns following a tandem skydive in anhedonic individuals are highly similar to patterns previously found in healthy individuals. Although replication is warranted, our findings tentatively suggest that a strong stress response that cannot be downregulated well predicts a decrease in self-esteem.

## Introduction

Free-fall experiences such as a skydive elicit fight-flight responses in virtually all individuals [1,2]. This is reflected in strong physiological stress responses (i.e., heart rate, cortisol levels and alpha-amylase levels) that return to normal not too long after completion of the skydive [3–5], as well as in psychological responses of intense fear during the free-fall [1,2], followed by euphoria after completion of the skydive [2,6].

There are indications that responses to stress and to reward interact; acute experimental stress has been found to reduce reward-related reactivity [7,8]. In one study, this effect was found only in individuals with strong stress responses [9]. The ability to experience reward has also been found to predict responses to stress and has been suggested to be of particular importance during recovery from stress [10].

Since a tandem skydive evokes both extreme stress and extreme reward, it may be an ultimate natural model to explore the complex relationship between acute stress responsiveness and reward responsiveness. It is particularly relevant to investigate these stress and reward responses in individuals with anhedonia, as anhedonia is characterized by dysfunctions in the reward system [11]. In the present study, we investigated individual differences in reactivity to and recovery from a tandem skydive in a sample of anhedonic young adults, and tested whether individual variations in trait and state characteristics within this group were associated with reactivity and recovery patterns. (Please note that we use the concepts trait and state to indicate the time-scale of the measurements, that is, from weeks to months (trait) versus daily or momentary experiences (state), without making claims about stability.) In addition, we investigated whether stress reactivity and recovery patterns were associated with changes in state characteristics from pre-skydive to post-skydive.

Anhedonia is characterized by low positive affect (PA), and is one of the two core symptoms of depression. Depression has been associated with worse stress recovery [12,13] and blunted stress reactivity [12,13], but also with higher stress reactivity [14]. A study that distinguished between PA and negative affect (NA) found that high arousal NA (i.e., angry, stressed, nervous, worried) predicted alpha-amylase increases, but only in adolescents with high average levels of these emotions; in the same study high arousal PA (feeling strong, active, excited) also predicted increases in alpha-amylase regardless of mean levels [15], suggesting that emotional arousal may be more relevant to alpha-amylase reactivity than valence (PA or NA). Another study found no association between pretest state PA and stress reactivity during a social stress test [16]. It is possible that these mixed findings are due to different stress reactivity measures, that is, heartrate variability [13,16], respiratory sinus arrythmia [16], cortisol [12,16] and alpha-amylase [14,15], but they may also be due to the type of stressor and the level of emotional arousal. Increased reactivity has been suggested in particular for novel and uncontrollable experiences [14] and for high arousal emotions [15]. As a tandem skydive is a prototypical example of a novel and uncontrollable experience evoking high arousal emotions, we expected that the severity of the affective problems would be positively associated with the reactivity to the skydive.

In addition to the findings described above, there is evidence that trait and state anxiety may affect stress reactivity, but the diversity of results [16–20] precludes expectations regarding the direction of the effects. Furthermore, there are indications that high state self-esteem [21] is associated with attenuated stress reactivity.

Apart from investigating trait and state characteristics as predictors of stress responses, it is also interesting to explore how stress response patterns in turn predict changes in state characteristics. Despite that several studies have explored state changes in response to experimentally induced stress, hardly any have explicitly examined how stress reactivity and recovery are associated with these changes. One study found that cortisol reactivity to a lab stress task was not related to pre- to post-stress changes in PA in healthy individuals [22]; another that heartrate reactivity to a social stress test was associated with pre- to posttest changes in PA and perceived control [16]. A third study found that that a fast cortisol recovery was associated with experiencing more PA right after a skydive [5]. Because in the latter study only PA assessed after the skydive was taken into account, it is unclear to what extent the association could be explained by pre-skydive PA. In the current study we assessed state anxiety, PA, and self-esteem both before and after the skydive, which enabled us to investigate whether stress reactivity and recovery were associated with changes in these state characteristics.

The main aim of our study was to investigate responses to the acute stress and euphoria evoked by a tandem skydive in young adults suffering from anhedonia who were novice to skydiving. We used salivary alpha-amylase levels as markers for physiological stress reactivity and recovery. Alpha-amylase is an enzyme that is secreted under autonomic regulation and has been found to be highly responsive to acute stress in humans [23,24]. As such, it has been frequently used as a biomarker for stress. As alpha-amylase not only rapidly increases in response to stress but, as opposed to cortisol, has also been found to decrease in response to relaxation [25], it is expected to function as an important marker for both stress reactivity and recovery. We examined (1) individual differences in reactivity to and recovery from a tandem skydive in a sample of anhedonic young adults; (2) whether trait depressive symptoms, anxiety, and PA (i.e., level of pleasure and reward responsiveness), and state anxiety, PA, and self-esteem prior to the skydive were associated with alpha-amylase reactivity and recovery patterns in response to a skydive; and (3) whether these reactivity and recovery patterns were associated with pre- to post-skydive changes in state anxiety, PA, and self-esteem.

## Materials and methods

### Participants

The present study is part of the larger intervention study “No Fun No Glory”, in which anhedonic young adults were given personalized lifestyle advice and exposed to tandem skydives as a possible mean to reduce their symptoms. A detailed description of the study protocol can be found in [26]. Participants were recruited from the general population by means of an online screening survey among 2,937 young adults (18–24 years). Inclusion criteria were persistent loss of pleasure and willingness to perform a skydive. It should be noted that, of the total sample that was screened, only 12.8% were unwilling to perform a tandem skydive (N = 376) and that these participants did not significantly (i.e., p > .05) differ from those who were willing (N = 1759; 59.9%), or from those who indicated ‘maybe’ (N = 802; 27.3%), on sex, age, trait PA, depressive symptoms (PHQ-9; [27]), and reward responsiveness (RR; [28]). Persistent loss of pleasure was measured with three items of the Domains of Pleasure Scale (DOPS; [29]). Participants needed to (1) score below the 25^th^ percentile on level of pleasure, (2) rate this level as less or much less than normal, and (3) report that this loss of pleasure was present for at least two months. Exclusion criteria were inability to keep an electronic diary three times a day; professional treatment for psychiatric problems; use of psychotropic medication; epilepsy; pregnancy; conditions that obstruct participating in a tandem skydive (i.e., loose prostheses; height of more than 2 meters; weight of more than 95 kg; inability to raise one’s legs 90 degrees; cardiovascular complaints or problems; and significant visual or hearing impairments); and experience with skydiving, bungee jumping, or base jumping. Hence, all participants were new to skydiving. The sample used in the present study consisted of 61 participants (M_age_ [SD] = 21.38 [1.98], 78.7% female), who were either randomly assigned to the intervention group who received a tandem skydive (N = 24) or initially assigned to another group and chose to participate in the tandem skydive themselves in a later phase of the study (N = 37). These groups did not differ in demographics (i.e., age, gender, BMI), nor in the level of anhedonia, depression, anxiety, or reward responsiveness measured at baseline, before the first (randomized) intervention, and before the second (free-choice) intervention, and also did not differ in alpha-amylase reactivity, recovery or mean level in response to the skydive (p > .05).

### Procedure

Participants who were eligible to participate in the intervention study received an information letter and were included in the study after providing their written informed consent. All participants filled out momentary assessments on their smartphone, three times a day with fixed 6h intervals for at least three months. Only data from the week before the skydive and the day of the skydive were used in the present study. Participants filled out monthly questionnaires at the start of the study and after two and three months. The tandem skydive always took place during weekends, at the skydive center Eelde-Hoogeveen. The tandem skydive was performed from a small turbine-powered aircraft (i.e., Cessna 207), at a height of 10,000 feet. Participants were safely attached to the tandem skydive instructor. The duration of the free fall to 5,000 feet was 30 to 40 seconds, the duration of the total skydive was around 5 minutes. Participants were instructed not to eat, drink (except water), smoke or eat chewing-gum in the two hours before the skydive. They had to be present at the skydive center at least half an hour before the skydive. Saliva samples were collected by means of cotton salivettes, which were administered at four time points: 15 minutes after the participants arrived at the skydive center, after they had received the instructions and were ready to board the plane, immediately after they had landed, and 20 minutes after they had landed. Participants were instructed to put the salivette in their mouth without touching it with their hands, and to keep it in their cheek, while occasionally chewing on it to let the cotton be absorbed by saliva for two minutes, as timed by the researcher that was in control of the procedure. The salivettes were immediately stored in a cooling box containing cooling elements. They were transported to Groningen and kept in a fridge for a maximum of two days, and then transported to the laboratory of the University Medical Center Groningen (UMCG). At the lab, the samples were centrifuged at 1000xg at 4°C for 2 minutes, aliquoted and stored at -80°C. The current study was registered in the Dutch Clinical Trial Register (NTR5498) and approved by the Medical Ethical Committee from the University Medical Center Groningen (no. 2014/508).

### Measures

#### Alpha-amylase

All alpha-amylase samples were analyzed in the same week, on four different days, at the general haematology and chemistry Lab in the UMCG, by means of an enzymatic colorimetric analysis, according to the International Federation of Clinical Chemistry (IFCC) method [30], on the Roche Modular P analyzer (Roche Diagnostics GmbH, Mannheim, Germany). Samples were defrosted overnight and centrifuged at 1520xg at room temperature for 3 minutes the next morning. All samples were diluted 1:100 with saline in an automatic onboard dilution step. Samples with alpha-amylase levels that were still too high after the automatic dilution were first diluted with saline 1:10 manually before the automatic dilution of 1:100, resulting in a total dilution of 1:1000. Results for the manually diluted samples were multiplied by 10. The total coefficients of variation were between 2.5% (for low levels of alpha-amylase) and 5.2% (for high levels of alpha-amylase) with mean inter assay coefficients of variation between 1.7% and 3.2%. We calculated the mean level of the alpha-amylase levels across the four assessments. For reactivity, we calculated the difference between T3 (i.e., first assessment after the skydive) and T2 (last assessment before the skydive) and divided this by the level at T2 to obtain the proportional increase in alpha-amylase compared to each individual’s level before the skydive (see Eq 1). For recovery, we calculated the difference between T4 (i.e. second assessment after the skydive) and T3 (first assessment after the skydive) and divided this by the level at T3 to obtain the proportional decrease in alpha-amylase compared to each individual’s level right after the skydive (see Eq 2).

$$Reactivity\;=\;\frac{\left(amylase\;T3-amylase\;T2\right)}{amylase\;T2}$$ (1)

$$Recovery\;=\;\frac{\left(amylase\;T4-amylase\;T3\right)}{amylase\;T3}$$ (2)

We used these measures of relative change for reactivity and recovery, because we were primarily interested in how much participants’ alpha-amylase levels changed from one assessment to the next relative to their own prior alpha-amylase level, and aimed to separate this within-subject effect from a possible confounding (between-subject) effect of mean alpha-amylase level, which is known to be highly variable across individuals.

#### Baseline assessments

Depressive and anxiety problems during the past six months were assessed at baseline with the Adult Self-Report (ASR), which is a standardized questionnaire of behavioral and emotional problems, which has been shown to have good reliability and validity [31]. We used the ASR depressive problems scale (14 items) and the anxiety problems scale (7 items) that are based on the Diagnostic and Statistical Manual of Mental Disorders, fourth edition (DSM-IV; [32]. For each item, answer categories are: 0 = ‘Not True’; 1 = ‘Somewhat or Sometimes True’; 2 = ‘Very True or Often True’. In our sample, Cronbach’s alpha was .78 for depressive problems and .78 for anxiety problems. Participants filled out the Domains and Dimensions of Pleasure Scale (DDOPS), in which we assessed pleasure during the past two weeks with a VAS ranging from “*I experience little pleasure*” to “*I experience a lot of pleasure*”. Reward responsiveness during the past two weeks was measured with the Reward Responsiveness scale [28], which consists of 8 items that are scored on a 4-point scale ranging from *strong disagreement* (1) to *strong agreement* (4). Cronbach’s alpha was .84.

#### Momentary assessments

In the momentary assessments participants reported their anxiety, PA and self-esteem. All items were rated on a Visual Analogue Scale (VAS), ranging from *not at all* to *very much*. Participants rated each item by moving a slider along the scale. The position of the slider was transformed into a score between 0–100. Anxiety was a combined measure of the items anxious and nervous (inter-item correlation .86). Positive affect (PA) was measured with six items, which were divided in low arousal PA (i.e., calm and relaxed; inter-item correlation .93), moderate arousal PA (i.e., joyful and cheerful; inter-item correlation .89), and high arousal PA (i.e., enthusiastic and energetic; inter-item correlation .88). We considered low, moderate and high arousal PA separately, because we expected that there may be a difference in how they would affect stress reactivity and recovery. To our best knowledge, this has not yet been tested. Self-esteem was measured with the item ‘I was pleased with myself’.

#### Covariates

Age [33], gender [34], BMI [35], smoking [36], alcohol use [33,36], physical activity [36], asthma medication [36], and time of day [35,37,38] may have an effect on alpha-amylase. At baseline, participants reported their age, gender, weight, and height. Weight and height were used to calculate BMI. Participants also reported whether they used medication and if so, which type. Reported medications were checked for being prescribed for asthma. Participants reported at each momentary assessment how many cigarettes and how many alcoholic beverages they consumed since the previous assessment. All cigarettes and alcoholic drinks consumed during the week before the skydive were summed into a total smoking and a total alcohol consumption score. Physical activity was measured with the momentary assessment item ‘I was physically active’, which was rated on a VAS ranging from *not at all* to *very much*. We included the last physical activity measure before the skydive (i.e., either the morning or afternoon assessment, depending on the time of the skydive). For time of day, we included the time of assessment of the second salivette (i.e., immediately before entering the plane). Time of day was calculated as the number of minutes past midnight.

### Strategy of analyses

#### Alpha-amylase patterns and descriptive statistics

First, we plotted the group-based mean alpha-amylase levels before (two assessments) and after (two assessments) the tandem skydive, as well as each individual secretion pattern. For the group-based plot as well as for all below-described statistical analyses we used SPSS version 25 [39]; for the individual plots we used R packages dyplr version 0.7.4 [40], ggplot2 version 2.2.1 [41], and cowplot version 0.9.2 [42], R version 3.4.3 [43]. Second, we calculated mean levels of and correlations among the trait and state measures, and alpha-amylase mean level, reactivity and recovery. Trait measures were based on the baseline assessments and state measures on the momentary assessments during the day before the skydive and on the day of the skydive. Because previous research found a diurnal pattern in alpha-amylase levels (i.e., levels increased during the day; [15]), we checked whether the timing of the alpha-amylase assessments was associated with mean levels.

#### Regression analyses

We used hierarchical regression models to examine whether trait depressive problems, anxiety problems, and PA (pleasure level and reward responsiveness) were associated with alpha-amylase reactivity and recovery. In addition, we checked whether the association between the predictors and recovery was moderated by reactivity by including the interaction between the predictors and reactivity in the model. All predictors were grand-mean centered before the interaction term was calculated. The same analyses were repeated for state levels of anxiety (i.e., anxious/nervous), PA (i.e., PA low arousal, PA moderate arousal, PA high arousal), and self-esteem, averaged over the day before the skydive. To examine whether reactivity and recovery were associated with a change in state levels from the evening before the skydive to the evening after the skydive, we conducted hierarchical regression analyses with state level at the evening after the skydive as dependent variable (i.e., anxiety, PA low arousal, PA moderate arousal, PA high arousal, self-esteem), and reactivity and recovery as predictors, while controlling for state level at the evening before (step 1). In a second step, we explored whether the effects of recovery on affect were moderated by reactivity by entering the interaction between reactivity and recovery. Again, predictors were grand-mean centered before computing the interaction term.

For all statistically significant findings (*p* < .05), we performed sensitivity analyses to check whether the results changed when adjusted for potential confounders. Since alpha-amylase reactivity and recovery may partly depend upon mean alpha-amylase levels, we also corrected for this variable in our sensitivity analyses.

## Results

### Alpha-amylase patterns

**Fig 1:**
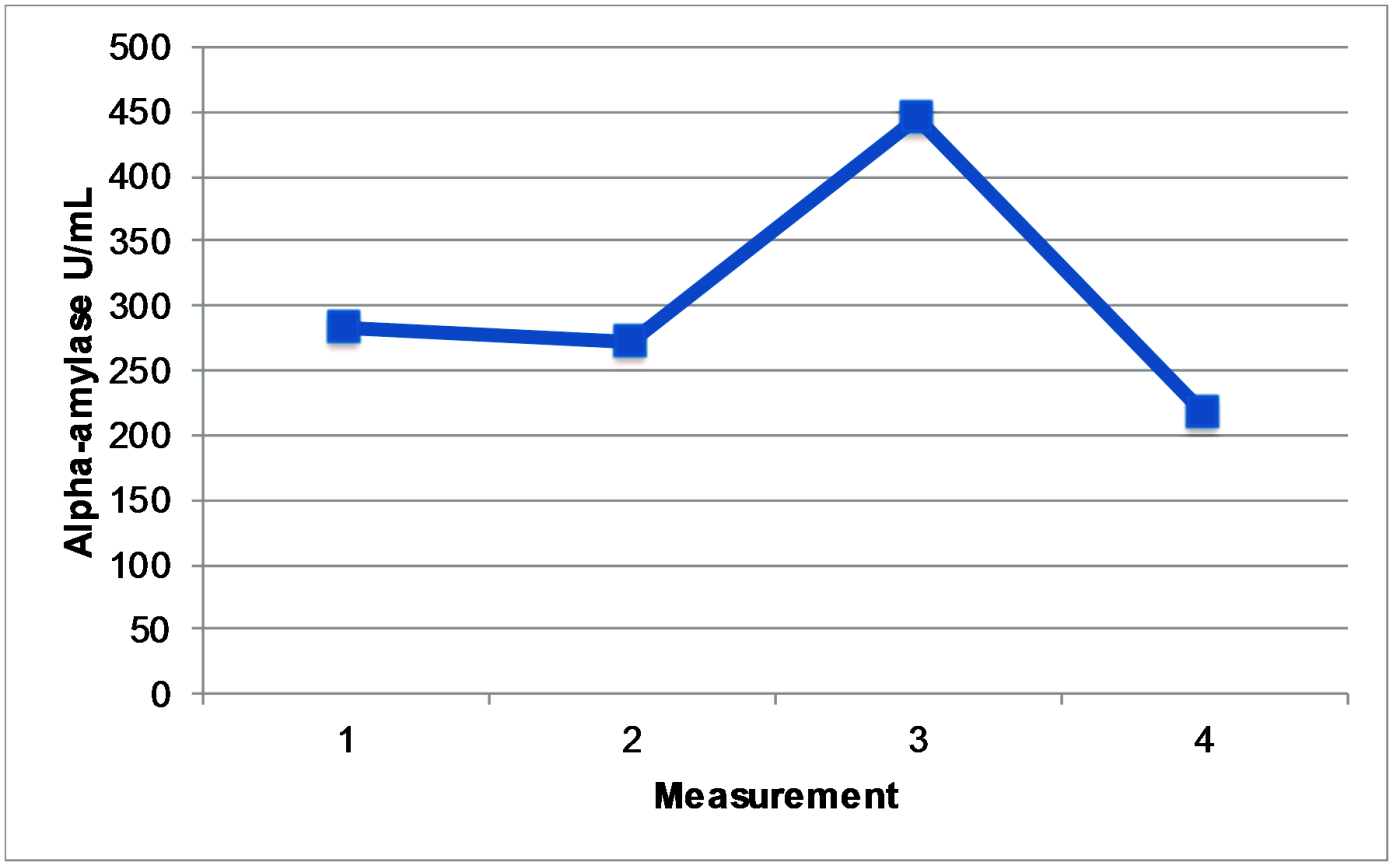
Plot mean alpha-amylase levels (U/mL) per measurement. The first measurement took place 15 minutes after the participant arrived at the skydive center, the second after they received the instructions and were ready to board the plane, the third immediately after they landed, and the fourth 20 minutes after they landed.

**Fig 2:**
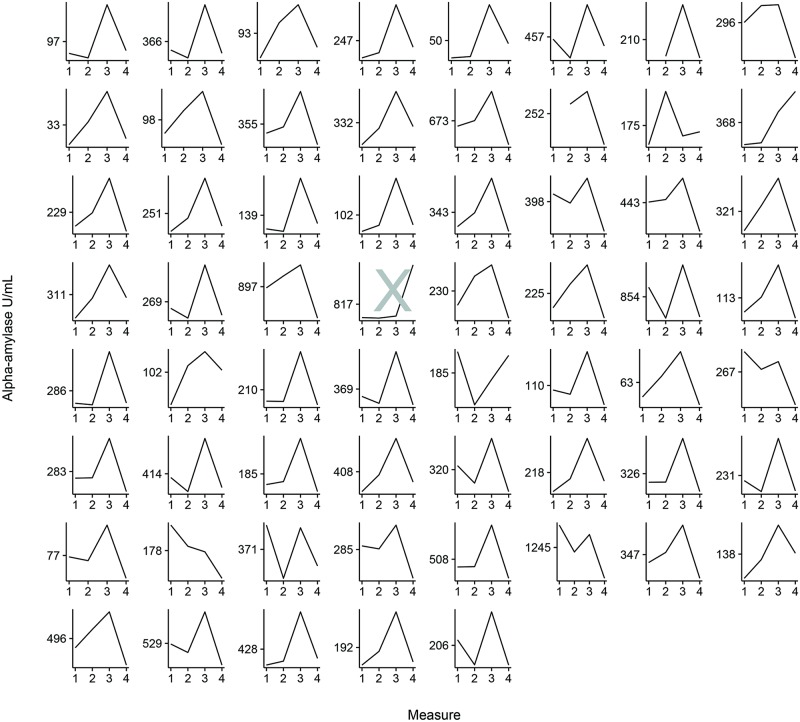
Individual plots alpha-amylase levels (U/mL) on four measurements surrounding the tandem skydive. The first measurement took place 15 minutes after the participant arrived at the skydive center, the second after they received the instructions and were ready to board the plane, the third immediately after they landed, and the fourth 20 minutes after they landed. The mean score of the four measurements is shown on the y-axis. The grey cross indicates a participant with an outlier on T4. This participant was removed prior to further analyses.

As can be seen in Fig 1, on average the alpha-amylase levels showed a strong increase between T2 and T3 (reactivity) and a strong decrease between T3 and T4 (recovery). Fig 2 shows that this pattern applied to most individuals. One participant, indicated by the grey cross in Fig 2, showed a suspiciously high alpha-amylase level at T4 combined with very low levels at T1-T3. We decided to discard this individual from further analyses. After removal of this outlier, 57 out of in total 60 participants showed an increase at T3 compared to T2 and an equal number of (partly different) participants showed decreasing alpha-amylase levels at T4 as compared to T3. Mean alpha-amylase levels per participant were highly variable, and ranged between 33 U/mL and 1245 U/mL.

### Descriptive statistics

Means of and correlations between study variables are depicted in Table 1. The proportional reactivity and recovery scores were moderately correlated, indicating that stronger reactivity is associated with stronger recovery. None of the affect measures were associated with mean alpha amylase levels, reactivity or recovery. The time at which the skydive took place was not associated with mean alpha-amylase levels (*r* = .12, p = .37), indicating that mean levels of participants skydiving in the afternoon were not higher than mean levels for participants skydiving in the morning.

**Table 1:** Descriptive statistics.

	Mean (SD)	Range	1	2	3	4	5	6	7	8	9	10	11	12	13	14	15	16	17	18	19	20	21
1. Average alpha-amylase	303.41 (211.89)	33.40–1244.62	-																				
2. Reactivity	0.77 (0.71)	-0.19–3.73	-.03	-																			
3. Recovery	0.47 (0.21)	-0.25–0.78	.15	.48**	-																		
4. Baseline depressive problems	0.79 (0.34)	0.07–1.57	-.15	-.02	-.10	-																	
5. Baseline anxiety problems	0.86 (0.44)	0.14–1.71	-.04	-.11	-.02	.60**	-																
6. Baseline pleasure	35.34 (13.93)	0.53–55.74	.09	-.01	-.20	-.39**	-.13	-															
7. Baseline reward responsiveness	25.97 (3.83)	10–32	-.06	.07	-.12	-.10	-.05	.26*	-														
8. State anxiety day before skydive	16.90 (13.38)	2.00–66.95	-.06	-.03	-.12	.24	.32*	-.09	.10	-													
9. State PA low arousal day before skydive	60.95 (14.81)	11.74–91.03	.14	-.00	.14	-.40**	-.44**	.17	.01	-.37**	-												
10. State PA moderate arousal day before skydive	58.75 (12.69)	10.74–93.01	.03	.09	.13	-.36**	-.41**	.16	.08	-.14	.73**	-											
11. State PA high arousal day before skydive	55.69 (14.92)	11.69–93.67	.04	.08	-.01	-.21	-.33*	.10	.15	.01	.62**	.87**	-										
12. State self-esteem day before skydive	52.22 (13.23)	9.73–92.44	.11	-.02	.04	-.41**	-.38**	.29*	.13	-.24	.65**	.77**	.74**	-									
13. State anxiety evening before skydive	17.19 (17.12)	.89–96.60	-.06	-.06	-.11	.21	.37**	-.04	.08	.87**	-.21	-.10	.10	-.19	-								
14. State PA low arousal evening before skydive	60.22 (17.56)	9.21–90.51	.07	.03	.17	-.42**	-.43**	.20	-.05	-.45**	.87**	.68**	.48**	.55**	-.46**	-							
15. State PA moderate arousal evening before skydive	60.05 (16.28)	9.49–94.05	.01	.14	.08	-.41**	-.39**	.25	.04	-.24	.56**	.84**	.64**	.66**	-.40**	.74**	-						
16. State PA high arousal evening before skydive	57.20 (16.65)	11.08–93.20	-.01	.04	-.15	-.30*	-.30*	.26	.15	-.03	.48**	.78**	.88**	.64**	-.07	.52**	.75**	-					
17. State self-esteem evening before skydive	52.24 (15.85)	18.46–93.77	-.05	-.00	-.04	-.36**	-.32*	.38**	.18	-.22	.42**	.64**	.57**	.85**	-.32*	.52**	.71**	.64**	-				
18. State anxiety evening after skydive	23.70 (22.85)	0.71–95.98	-.13	-.04	-.06	.23	.26	-.24	.00	.69**	-.25	-.11	.02	-.15	.68**	-.39**	-.31*	-.11	-.27	-			
19. State PA low arousal evening after skydive	62.89 (16.96)	5.54–92.21	.11	-.01	-.02	-.32*	-.27*	.32*	.19	-.22	.69**	.54**	.47**	.35**	-.03	.72**	.49**	.55**	.29*	-.50**	-		
20. State PA moderate arousal evening after skydive	68.72 (13.39)	15.61–95.89	-.02	-.00	-.06	-.31*	-.19	.25	.23	-.00	.57**	.71**	.74**	.57**	.14	.48**	.49**	.71**	.42**	.00	.54**	-	
21. State PA high arousal evening after skydive	65.85 (14.90)	17.29–95.75	.03	-.11	-.08	-.12	-.06	.14	.22	.16	.42**	.55**	.68**	.45**	.26	.26	.31*	.58**	.30*	.23	.32*	.83**	-
22. State self-esteem evening after skydive	63.58 (17.46)	8.39–95.97	.05	.03	.04	-.30*	-.17	-.02	.16	.02	.34*	.43**	.46**	.43**	.10	.23	.25	.37**	.26	.08	.27	.74**	.74**

*** *p* < .001,

** *p* < .01,

* *p* < .05.

PA = positive affect.

### Trait and state measures as predictors of alpha-amylase patterns

First, we explored associations between trait and state measures and reactivity and recovery (Table 2). Trait depressive and anxiety problems were entered in the model simultaneously and both trait PA measures were also entered in one model simultaneously. For the state measures, this was not possible due to multicollinearity issues. No direct associations were found between the trait and state measures and reactivity or recovery.

**Table 2:** Regression analyses for trait and state predictors on alpha-amylase reactivity and recovery.

		Alpha-amylase							
		Reactivity				Recovery			
		B	*β*	*p*	*R*^*2*^	B	*β*	*P*	*R*^*2*^
**Trait models**	Depressive problems	0.23	0.11	.54		-0.09	-0.14	.43	
	Anxiety problems	-0.32	-0.19	.28	.02	0.03	0.06	.75	.01
	Pleasure	-.01	-.12	.39		-.00	-.20	.14	
	Reward responsiveness	.02	.09	.49	.02	-.01	-.10	.47	.05
**State models**	Anxiety	-0.00	-0.04	.79	.00	-0.00	-0.12	.39	.01
	PA low arousal	0.00	0.00	.98	.00	0.00	0.14	.29	.02
	PA moderate arousal	0.01	0.10	.47	.01	0.00	0.14	.30	.02
	PA high arousal	0.00	0.08	.54	.01	-0.00	0.00	.99	.00
	Self-esteem	-0.00	-0.02	.88	.00	0.00	0.04	.79	.00

PA = positive affect. Please note that all state predictors were analyzed in separate regression models, due to high inter-correlations.

Reactivity did not modify the associations between trait and state measures and recovery; all interaction effects were non-significant (depressive problems: *β* = -0.03, *p* = .80, anxiety problems: *β* = 0.02, *p* = .87, ΔR^2^ for full model = .00, level of pleasure: *β* = 0.02, *p* = .87, reward responsiveness: *β* = -0.06, *p* = .67, ΔR^2^ for full model = .00, state anxiety: *β* = -0.01, *p* = .96, ΔR^2^ = .00, PA low arousal: *β* = -0.01, *p* = .93, ΔR^2^ = .00, PA moderate arousal: *β* = 0.01, *p* = .97, ΔR^2^ = .00, PA high arousal: *β* = -0.01, *p* = .94, ΔR^2^ = .00, and self-esteem: *β* = 0.15, *p* = .28, ΔR^2^ = .02).

### Alpha amylase reactivity and recovery as predictors of changes in anxiety, PA and self-esteem

**Fig 3:**
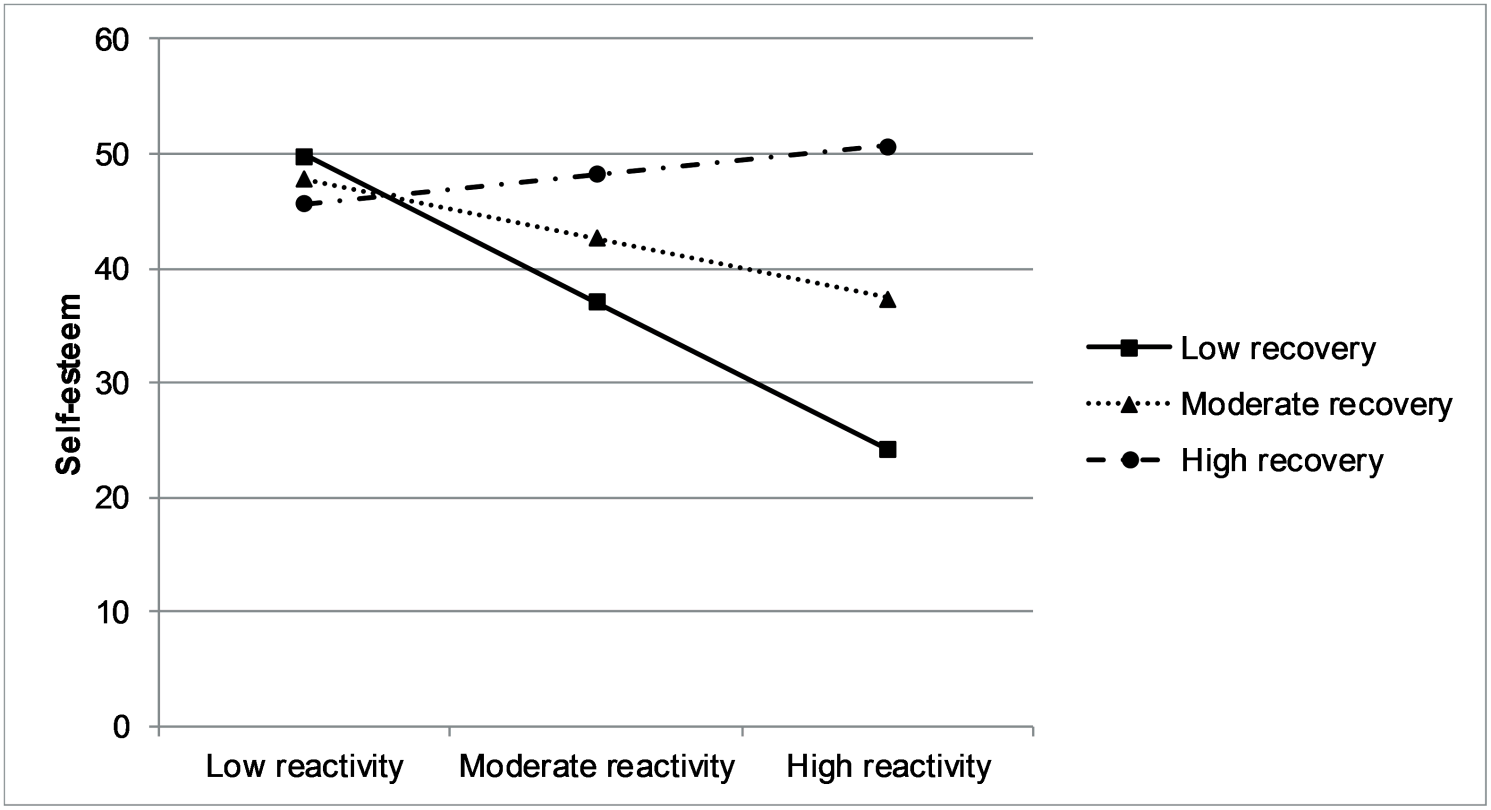
Interaction between reactivity and recovery on self-esteem.

Neither alpha-amylase reactivity nor recovery predicted anxiety, PA and self-esteem reported at the evening after the skydive (Table 3). The interaction between reactivity and recovery was significant for self-esteem (*β* = 0.46, *p* = .03, ΔR^2^ = .10). As can be seen in Fig 3, when individuals showed low reactivity, they experienced high levels of self-esteem after the skydive, irrespective of their level of recovery. In contrast, self-esteem was low for individuals who show high levels of reactivity and low levels of recovery. No significant interactions between reactivity and recovery were found for anxiety (*β* = - 0.18, *p* = .29, ΔR^2^ = .01), PA low arousal (*β* = 0.10, *p* = .53, ΔR^2^ = .00), PA moderate arousal (*β* = 0.34, *p* = .08, ΔR^2^ = .05), or PA high arousal (*β* = 0.10, *p* = .61, ΔR^2^ = .00).

**Table 3:** Regression analyses for alpha-amylase reactivity and recovery on state anxiety, PA, and self-esteem after the skydive.

	Anxiety				PA low arousal				PA moderate arousal				PA high arousal				Self-esteem			
	B	*β*	*p*	ΔR^2^	B	*β*	*p*	ΔR^2^	B	*β*	*p*	ΔR^2^	B	*β*	*p*	ΔR^2^	B	*β*	*p*	ΔR^2^
Evening before	0.78	0.69	.00		0.65	0.75	.00		0.42	0.50	.00		0.51	0.58	.00		0.30	0.26	.09	
Reactivity	-0.38	-0.02	.90		0.99	0.05	.67		0.15	0.01	.96		-2.51	-0.13	.36		0.89	0.04	.82	
Recovery	3.87	0.04	.75	.00	-13.18	-0.18	.15	.02	-10.76	-0.16	.29	.02	-0.19	-0.00	.99	.02	1.27	0.01	.93	.05

PA = positive affect. Evening before = controlled for level on evening before the skydive. ΔR^2^ represents the change in explained variance when reactivity and recovery were added as predictors to the model.

### Sensitivity analyses

In a final step, we checked for statistically significant findings (*p* < .05) whether controlling for the previously mentioned covariates affected our results (for detailed results see S1 Table available online). The reported effect that was significant remained significant after entering the covariates.

## Discussion

The main aim of the present study was to investigate reactivity and recovery patterns in alpha-amylase levels evoked by a tandem skydive. Results indicated that, although mean alpha-amylase levels across the measures greatly differed among individuals, the reactivity and recovery patterns were highly similar. We found no associations between trait or state measures and reactivity or recovery. Finally, the association between reactivity and self-esteem after the skydive depended on the level of recovery: individuals who showed high levels of reactivity in combination with low levels of recovery experienced the lowest levels of self-esteem.

While the mean alpha-amylase levels of our participants greatly differed, the reactivity and recovery patterns were highly comparable (see Fig 2); most participants were highly reactive to the skydive (increase in alpha-amylase levels) and showed a quick recovery afterwards (decrease in alpha-amylase levels). Mean levels of alpha-amylase in our study (i.e., 303.41 U/mL) were, as expected, high compared to reported alpha-amylase levels in studies without an acute stressor (i.e., 224.1 U/mL for depressed and 173.9 U/mL for non-depressed individuals [44]. Similar to a previous skydiving study in non-anhedonic individuals [3], in our study the highest mean alpha-amylase levels were measured right after landing. However, the mean alpha-amylase levels directly after the skydive were rather low in our sample (444 U/mL), compared to levels found in Chatterton’s study (900 U/mL, [3]. This may be due to the fact that the participants in Chatterton’s study were responsible for opening the parachute during the tandem skydive themselves, which may have added to their stress levels; it may also be partly due to the anhedonic symptoms of our study participants. Chatterton and colleagues reported a 40% decrease in alpha-amylase between the sample collected immediately after landing and the one collected 15 minutes later. This pattern of stress recovery closely matched the pattern found in our anhedonic young adults who showed an average decrease in alpha-amylase of 49% between the sample collected after landing and the sample collected 20 minutes later. Overall, similarities between salivary alpha-amylase reactivity and recovery patterns we report in the present study and those reported in studies on non-anhedonic samples suggest that (sub-clinical) anhedonic symptoms may not largely impact the reactivity to and recovery from ultimate thrill experiences such as a tandem skydive.

Previous research has indicated that trait depression and anxiety may be associated with higher alpha-amylase levels [44,45] and trait depression with increased alpha-amylase reactivity in response to an acute stressor[14], but we did not find these associations in the present study. These diverging findings might be due to the fact that we examined these associations within an anhedonic sample, whereas most previous studies compared stress responses between healthy individuals and psychiatric populations [14,45]. We also expected that reward responsiveness would be associated with stress responses to the skydive, particularly that higher reward responsiveness would facilitate stress recovery [10], but we did not find this. Speculatively, perhaps differences in reward responsiveness in daily life situations, as measured with the Reward responsiveness Scale, do not apply to the extreme feelings of euphoria experienced after a skydive. If this is indeed the case slightly less extreme stressors may be more appropriate to investigate if reward responsiveness is related to individual differences in stress recovery. Similarly, levels of depression and anxiety may explain responses to stress in daily life situations, but perhaps not in response to the ultimate stress experience of a skydive. However, further research is needed to explore these potential explanations.

Although the effect was small and should be interpreted with caution, results suggested that the combination of reactivity and recovery rates might affect self-esteem. Individuals who experienced either low reactivity or high recovery showed high post-skydive self-esteem, but individuals who combined high reactivity with low recovery experienced relatively low self-esteem after the skydive. This positive effect of high recovery is partly in line with a previous study in skydivers, which found that greater happiness after the skydive was associated with faster recovery in cortisol [5]. Whereas the direction of effects was unclear in this previous study, we found that high recovery was prospectively associated with a positive change in self-esteem. We only found this effect for self-esteem and not for our other mood measures. This could imply that being able to downregulate your stress levels after such an intense stress experience leads to a boost in self-confidence, but not to a boost in mood per se. We acknowledge that this finding was small (*p* = .03), and would not survive multiple testing correction. Hence, it could be a false positive finding and should be interpreted with caution. On the other hand, the effect size (*β* = 0.46, explained variance = 10%) was not that small and given our rather small sample size, lack of power may also explain why this finding would not survive correction for multiple testing.

### Strengths and limitations

The present study had multiple strengths, including the extreme nature of the stressor, which is a novel and uncontrollable experience that elicits a fight-flight response in all individuals. Further, we included intensive momentary measurements before and after the skydive, and hence were able to obtain a detailed picture of affective experiences around the skydive. However, the results of this study should be interpreted in the context of some limitations. Given that we only included anhedonic individuals, our results may not be generalizable to the general population. Because of the lack of a control group we were also unable to compare stress responses to the skydive between anhedonic and non-anhedonic individuals in the current study, and thus were limited to comparing our findings for anhedonic individuals to findings for healthy individuals reported in other studies. Further, because of the unequal sex distribution (21.7% males) and small sample size, we were not able to examine sex differences in the associations. Another limitation concerns the alpha-amylase sampling. The first (baseline) saliva sample was collected when participants arrived at the skydive center and may have been stressed already. Therefore, we could not examine whether participants recovered to their actual baseline levels. Further, for practical purposes and convenience for the participants we used salivettes to assess alpha-amylase levels, a method that has some limitations [46]. One of the main limitations is that chewing affects the flow rate of saliva, which may affect the alpha-amylase concentration. Therefore, a passive drooling technique might have been better. Finally, we did not assess how participants experienced the skydive directly and therefore had to rely on how they felt before and after the skydive.

### Conclusion

In sum, we showed that alpha amylase reactivity and recovery patterns in response to a skydive in anhedonic individuals were highly consistent with patterns previously found in healthy individuals. Within an anhedonic sample, trait depressive and anxiety problems, trait PA, and state anxiety and PA were not associated with reactivity and recovery patterns. Finally, although replication in larger samples is warranted, our results tentatively suggest that in individuals with high stress reactivity, the ability to downregulate one’s stress levels after a skydive was related to a boost in self-esteem, but not to mood in general.

## Supporting information

S1 Table Regression analyses for alpha-amylase reactivity and recovery on affect after the skydive, full model including all covariates. *Note*. BMI = Body Mass Index.
(PDF)
