# Supplementary material for: Alpha-amylase reactivity and recovery patterns in anhedonic young adults performing a tandem skydive
Source: PLoS One. 2018 Sep 24;13(9):e0204556. doi: 10.1371/journal.pone.0204556 (PMC6152985; doi:10.1371/journal.pone.0204556)
Supplement: S1 Table — Note. BMI = Body Mass Index. (PDF) [file pone.0204556.s001.pdf]

Supplemental material

Alpha-amylase reactivity and recovery patterns in anhedonic young adults performing a tandem skydive

By Vrijen, Van Roekel & Oldehinkel, *PLOS ONE*

Table S1. Regression Analyses for Alpha-Amylase Reactivity and Recovery on Affect After the Skydive, full model including all covariates.

	Self-esteem		
	B	*β*	*p*
Self-esteem evening before skydive	0.23	.20	.203
Reactivity	-6.32	-.27	.263
Recovery	32.71	.37	.106
Reactivity * Recovery	51.39	.45	.045
Mean amylase level	-0.01	-.13	.461
Age	3.02	.34	.025
Gender	4.76	.11	.538
BMI	-0.99	-.17	.330
Average number of cigarettes (week before skydive)	0.16	.13	.539
Average number of alcoholic beverages (week before skydive)	0.19	.09	.633
Physical activity (assessment before the skydive)	-0.28	-.20	.238
Asthma medication	-3.05	-.05	.724
Time of day	0.02	.16	.282

Note. BMI = Body Mass Index
